# *Salmonella* *enterica* Serotype Enteritidis Phage Type 4b Outbreak Associated with Bean Sprouts

**DOI:** 10.3201/eid0804.010213

**Published:** 2002-04

**Authors:** Yvonne T.H.P. van Duynhoven, Marc-Alain Widdowson, Carolien M. de Jager, Teresa Fernandes, Sabine Neppelenbroek, Winette van den Brandhof, Wim J.B. Wannet, Jan A. van Kooij, Henk J.M. Rietveld, Wilfrid van Pelt

**Affiliations:** * National Institute of Public Health and the Environment, Bilthoven, the Netherlands; † Inspectorate for Health Protection and Veterinary Public Health, the Hague, the Netherlands; ‡ Inspectorate for Health Protection and Veterinary Public Health, Region East, Zutphen, the Netherlands

**Keywords:** Salmonella, outbreak investigation, bean sprouts, Enter-net

## Abstract

In November 2000 in the Netherlands, an outbreak of *Salmonella enterica* serotype Enteritidis phage type 4b was investigated. Eating bean sprouts was the only exposure associated with *S.* Enteritidis pt 4b infection (matched odds ratio 13.0, 95% confidence interval 2.0-552.5). Contaminated seeds were the most likely cause of contamination of the sprouts. The sprout grower applied a concentration of hypochlorite solution that was too low for seed disinfection.

*Salmonella* is one of the most common causes of bacterial gastroenteritis in the Netherlands and is often implicated in foodborne outbreaks (*1*,*2*). Raw or undercooked meat, eggs, raw milk, and especially poultry are well-known vehicles for transmission of *Salmonella* spp. However, fresh produce, such as lettuce and unpasteurized apple or orange juice, has also caused outbreaks (*3*–*5*). In the last decade, multiple outbreaks of *Salmonella* spp. (and Shiga-toxin-producing *Escherichia coli* O157:H7) linked to seed sprouts have occurred throughout the world (*6*–*8*). In the Netherlands, a recent study of sprouted seed products showed that 0.9% of 666 samples of bean sprouts contained *Salmonella* spp. (*9*). We report the first confirmed outbreak of *Salmonella enterica* serotype Enteritidis phage type (pt) 4b associated with bean sprouts in our country.

## The Study

On November 27, 2000, an outbreak detection algorithm identified a cluster of 12 cases with *S.* Enteritidis pt 4b in data from the National Reference Center for Salmonella at the National Institute of Public Health and the Environment (RIVM). This reference system covers 64% of all laboratory-confirmed salmonellosis in the country. Since the implementation in 1997 of the Colindale phage-typing system, no cases of this phage type had been reported. In the week before these cases were identified, the same phage type was found by the reference laboratory for an isolate detected during a quality control inspection of one batch of bean sprouts harvested on October 26, 2000 (typed as 4b on November 23).

In the first week of the outbreak, eight of the initial patients were interviewed by using a hypothesis-generating questionnaire adapted from the “*Salmonella* Trawl” (an existing questionnaire of the Communicable Disease Surveillance Centre in England). Three foods—chicken (reported by all), eggs (seven reports), and bean sprouts (six reports)—emerged as possible risk factors for infection. A case-control study was started on November 30.

We defined a case as diarrhea (>3 loose stools in a 24-hour period) after November 1, 2000, with a stool specimen positive for *S.* Enteritidis pt 4b. Secondary cases were included to measure the magnitude of the outbreak but excluded from the case-control study. For each case, one control was found by searching for persons by street name in a web-based phone book, matching for neighborhood, age group (<17, 18-40, >40 years), and sex.

Patients and controls were interviewed by telephone. The questionnaire addressed clinical manifestations (patients only), contacts with other symptomatic persons, and food consumption. Exposure factors were collected for the 7-day period before onset of illness for both patients and controls. A matched univariate analysis in Epi-Info 6.04 (Centers for Disease Control and Prevention, Atlanta, GA) was performed with maximum likelihood techniques to estimate odds ratios and exact confidence intervals (CI). To control for confounding, conditional logistic regression (exact method) was done in LogExact (Cytel Corp, USA.).

The sprout producer contacted the Inspectorate for Health Protection and Veterinary Public Health (hereafter referred to as Food Inspection Service) on November 30. The same day the company was inspected. Operational hygiene and cultivation procedures were reviewed. Three 25-g samples were taken from bean sprouts harvested that day; environmental samples were also obtained in case of air contamination during growth. All samples were tested for *Salmonella.*

Once it was determined by the epidemiologic study that bean sprouts were the suspected food source, patient data about the sales outlets for the sprouts were supplied to the Food Inspection Service. That agency then checked invoices from these businesses to identify the producer. No traceback was performed to the seed distributors of the implicated sprout producer.

The Netherlands participates in Enter-net, an international surveillance network for *Salmonella* and Verocytotoxin-producing *Escherichia coli* O157 infections, funded by the European Commission (*10*). All participants were informed about the cases and requested to forward information on recent cases of *S.* Enteritidis pt 4b or foodstuffs contaminated with this type. By this cross-the-border case-finding, international problems could be identified. In addition, the international database was reviewed for this specific phage type for the period 1998-2000.

A total of 27 cases were identified in the Netherlands. In addition, a second *Salmonella* isolate from bean sprouts was received from the same sprout producer. It had been harvested on November 15 and typed as 4b on November 30. Patients were found in 7 of the 12 provinces. The 26 primary cases had dates of onset from November 3 through November 24 (Figure). The last patient’s case was classified as secondary.

**Figure:**
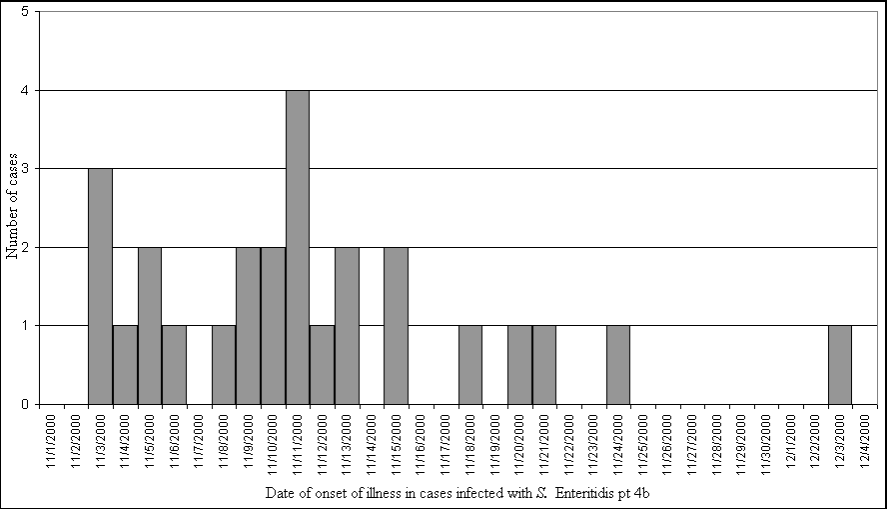
Epidemic curve of 27 identified cases (including 1 confirmed secondary case) in outbreak of *Salmonella enterica* serogroup Enteritidis phage type 4b, the Netherlands, November–December 2000

Of the patients, 67% were females. Ages ranged from 1 to 74 years, with 12 (44%) children <10 years of age. The median duration of illness was 8 days (range 5->25). The most frequently reported symptoms were diarrhea (100%; 56% bloody), abdominal cramps (92%), fever (76%), vomiting (40%), and headache (40%). Four female patients (16%) were admitted to the hospital for 3 to 7 days.

Data were collected for 24 matched pairs. The median age difference was 4 years in a pair (control older than the patient). In the univariate analysis, bean sprouts, chicken, tomatoes, celery, peppers, and onions were associated with illness at the 0.2 significance level (Table). Bean sprouts were the only variable associated with illness that was significant at the 0.05 level (matched odds ratio =13.0; CI 95% 2.0-552.5).Fifty-eight percent of patients recalled eating bean sprouts (Table). For seven patients who recalled the exact dates they ate the sprouts, the incubation period was 1 to 6 days (median 2 days). Of controls, 8% reported eating bean sprouts.

**Table:** Matched univariate analysis (24 pairs) of the association between *Salmonella enterica* serotype Enteritidis phage type 4b infection and various food items, the Netherlands, November–December, 2000

Food item	Exposed case, unexposed control No. of pairs	Unexposed case, exposed control No. of pairs	Matched OR^a^ (CI)^b^	% Cases exposed
Chicken	8	2	3.0 (0.8-38.7)	71
Beef	2	5	0.4 (0.04-2.4)	71
Fish	6	3	2.0 (0.4-12.4)	54
Eggs	4	2	2.0 (0.3-22.1)	79
Tomato	9	3	3.0 (0.8-17.2)	71
Lettuce	5	8	0.6 (0.2-2.2)	50
Alfalfa sprouts	1	0	Undefined	4
Cabbage	5	5	1.0 (0.2-4.4)	38
Bean sprouts	13	1	13.0 (2.0-552.5)	58
Cucumber	6	4	1.5 (0.4-7.2)	63
Paprika	6	4	1.5 (0.4-7.2)	54
Peppers	4	0	Undefined	17
Onion	9	3	3.0 (0.8-17.2)	67
Carrots	6	8	0.8 (0.2-2.5)	46
Parsley	6	2	3.0 (0.5-30.4)	38
Celery	7	2	3.5 (0.7-34.5)	29

^a^Maximum Likelihood Estimate of odds ratio (OR). ^b^Exact 95% confident limits for Maximum Likelihood Estimate of OR.

On the day the cluster was detected, the company that submitted the contaminated sprouts to the central laboratory was contacted and voluntarily took several measures 2 days later, including intensified testing and removal of the raw materials from which the contaminated sprouts were grown. In addition, as a routine practice, the room from which the contaminated sprouts were grown (separate rooms were used for each batch) was emptied, cleaned with a high-pressure equipment**,** and disinfected. No new cases of *S.* Enteritidis pt 4b have been reported since. The producer distributed sprouts almost nationwide and probably also exported to Germany. The Food Inspection Service’s inspection the next day, November 30, demonstrated that Hazard Analysis and Critical Control Points systems were used for both disinfection of the facility and cultivation of the sprouts. For irrigation, the producer had a permit for a private water system, which was checked daily for microbiologic quality. The Food Inspection Service found no irregularities in the recorded microbiologic data in the days before harvest of the contaminated sprouts. None of the employees in the production facility had been ill before or at the time the contaminated sprouts were harvested. All product and environmental samples taken by the Food Inspection Service were negative for *Salmonella*. At the implicated production facility, every new batch of seeds and samples from each batch of sprouts harvested each day was routinely tested for *Salmonella* spp. Test results are available after 5 days (the end of the shelf life for sprouts). So far, no seeds have been positive for *Salmonella*; however, of the annually tested batches of sprouts, 0.5% were positive. The producer applied seed disinfection with approximately 5 ppm hypochlorite solution during presoaking.

Fourteen patients reported the places where they bought the bean sprouts. For seven, the invoices could be checked (starting December 12) and were traced back to the same sprout producer that was inspected.

Information from Enter-net participants arrived within a few days after the cluster was detected. No country reported a recent increase in this phage type. In the Enter-net database, 15 (0.04%) of 33,773 and 24 (0.09%) of 26,336 *S.* Enteritidis isolates from 10 countries that were phage typed according to the Colindale system were type 4b in 1998 and 1999, respectively. In 2000, this was 139 (0.56%) of 24,961, mainly from Germany (57 since August), from our Dutch outbreak (27 isolates) and to a lesser extent from England and Wales (20 since June).

## Conclusions

This outbreak of *S.* Enteritidis pt 4b was identified following routine surveillance and prompt investigation. This relatively small outbreak was noticed because of the unusual phage type 4b; outbreaks caused by more common types would likely be easily missed. Since only a minority of patients with gastroenteritis are known to seek medical care and fewer still have their cases laboratory confirmed, the real number of affected persons in this outbreak is likely to be several hundred.

Both the detection of the same, rare type of *Salmonella* during routine control procedures for the product and the epidemiologic study incriminated bean sprouts as the cause of the outbreak. The fact that not all patients reported eating bean sprouts may be due to recall bias, since interviews took place 16 to 32 days after the onset of illness and sprouts are often served inconspicuously in salads, sandwiches, and other meals (*11*,*12*). Other explanations might be secondary transmission and cross-contamination of other food items.

The predominance of women, the broad age category, and the wide geographic distribution correspond with previous sprout-associated outbreaks (*7*,*8*). In >60% of households, the confirmed case-patient was the only one who had symptoms, even though some entire families ate the sprouts. The low attack rate in these households might reflect a low-level contamination of the sprouts or, more likely, a high-level, but heterogeneous contamination, i.e., high numbers of *Salmonella* in sprouts grown from a contaminated seed and little or no *Salmonella* in the remainder.

Based on the 20-day interval between the *Salmonella*-positive harvests of bean sprouts and lack of evidence for other sources of contamination, seeds were the most likely cause of contamination, as in most sprout-associated outbreaks (*9*,*13*). Although, the seeds tested negative for *Salmonella* in the routine control operations of the producer, testing is probably ineffective, as contamination may be intermittent and low level (*7*,*11*).

To date, no single treatment has been demonstrated to completely eliminate pathogens without affecting germination of the sprouts (*7*). Therefore, research on methods to reduce or eliminate pathogens is still ongoing (*7*,*13*). The sprout grower implicated in our outbreak applied seed disinfection with 5 ppm hypochlorite solution; recommended concentrations range from 2,000 to 20,000 ppm (*7*,*13*).

To monitor trends and detect producers with above-average contamination rates, incorporating the investigation of sprouted seed products in the routine program of food law enforcement in the Netherlands might be considered. As a result of the outbreak, all sprout-producing companies are being investigated by the Food Inspection Services; sprouts are tested for *Salmonella* spp*, Listeria monocytogens,* and *Bacillus cereus;* and hygiene procedures and disinfection treatment practices are reviewed.

This outbreak also alerted the Netherlands to the fact that the country has no legal basis to stop distribution of known *Salmonella*-contaminated sprouts. Although the law states that food products should not contain pathogenic microorganisms in 25-g samples of the product, an explicit exception is made for raw, unprocessed foodstuffs. This exception is made because it is assumed that raw foodstuffs will be processed before consumption, eliminating microorganisms. However, sprouting products are generally eaten raw or undergo only a mild or rapid heat treatment, such as stir-fry. This concept was brought to the attention of the Minister of Health.

During the outbreak investigation, Enter-net proved a rapid network for European communication on the occurrence of the specific phage type. Additionally, this database demonstrated an increase in the outbreak phage type in at least one other country in 2000, Germany; this increase is being investigated.

Finally, seed sprouting products entering the retail market still might contain pathogenic microorganisms. Thus, persons who are at increased risk for complications, such as young children, the elderly, the immunocompromised, and the chronically ill, should avoid eating these products. Many Dutch consumers may be unaware that sprouts, usually considered a healthy food, can cause foodborne illness. To make them aware, publicity efforts are needed.
